# Hydrogen Peroxide–Inducible Clone-5 Regulates Mesangial Cell Proliferation in Proliferative Glomerulonephritis in Mice

**DOI:** 10.1371/journal.pone.0122773

**Published:** 2015-04-02

**Authors:** Ariunbold Jamba, Shuji Kondo, Maki Urushihara, Takashi Nagai, Joo-ri Kim-Kaneyama, Akira Miyazaki, Shoji Kagami

**Affiliations:** 1 Department of Pediatrics, Institute of Health Bioscience, The University of Tokushima Graduate School, Tokushima, Japan; 2 Department of Biochemistry, Showa University School of Medicine, Tokyo, Japan; INSERM, FRANCE

## Abstract

Hydrogen peroxide-inducible clone-5 (Hic-5) is a transforming growth factor (TGF)-β1-inducible focal adhesion protein. We previously demonstrated that Hic-5 was localized in mesangial cells and its expression was associated with glomerular cell proliferation and matrix expansion in human and rat glomerulonephritis (GN). In the present study, we first assessed the role of Hic-5 in mesangioproliferative GN by injecting Habu venom into heminephrectomized wild type (Hic-5+/+) and Hic-5-deficient (Hic-5-/-) mice. Hic-5+/+ GN mice exhibited glomerular cell proliferation on day 7. Surprisingly, glomerular cell number and Ki-67-positive cells in Hic-5-/- GN mice were significantly greater than those in Hic-5+/+ GN mice on day 7, although the number of glomerular apoptotic cells and the expression of growth factors (platelet-derived growth factor-BB and TGF-β1) and their receptors were similarly increased in both Hic-5+/+ and Hic-5-/- GN mice. In culture experiments, proliferation assays showed that platelet-derived growth factor-BB and TGF-β1 enhanced the proliferation of Hic-5-/- mesangial cells compared with Hic-5+/+ mesangial cells. In addition, mitogenic regulation by Hic-5 was associated with altered and coordinated expression of cell cycle-related proteins including cyclin D1 and p21. The present results suggest that Hic-5 might regulate mesangial cell proliferation in proliferative GN in mice. In conclusion, modulation of Hic-5 expression might have a potential to prevent mesangial cell proliferation in the acute mitogenic phase of glomerulonephritis.

## Introduction

Progressive glomerular diseases are generally characterized by increased cell proliferation and extracellular matrix (ECM) accumulation, both of which lead to glomerulosclerosis [1]. Glomerulosclerosis, an irreversible pathological change, is a final common pathway of injured glomeruli in all progressive glomerular diseases including IgA nephropathy and diabetic nephropathy [2,3]. Mesangial cell (MC) proliferation, the representative initial hallmark of glomerular injury, generally precedes glomerulosclerosis and then often persists during the progressive course of glomerular diseases. In contrast, MC proliferation might be resolved in some types of glomerulonephritis (GN) including post-streptococcal glomerulonephritis in human or rat anti-Thy1 GN [4]. Thus, modulation of MC mitogenic activity might be important for determining whether glomerular injury progresses to advanced ECM accumulation or recovers from transient MC proliferation. However, the mechanisms of MC proliferation after glomerular injury remain poorly understood. There have been studies on these mechanisms from the perspective of cytokines. Isaka *et al*. demonstrated that platelet-derived growth factor (PDGF)-BB affected cell proliferation rather than ECM accumulation, and transforming growth factor (TGF)-β affected the latter rather than the former, suggesting that both growth factors play a role in glomerulosclerosis [5]. Barnes and Abboud suggested that PDGF-BB is a potent stimulator of MC proliferation, whereas TGF-β has potent antiproliferative effects and is a negative regulator of MC proliferation [6]. These findings indicate that TGF-β and its related molecules might play a role in the regulation of MC proliferation and ECM accumulation while PDGF-BB enhances MC proliferation after glomerular injury.

A growing body of evidence indicates that cell adhesion to ECM via integrins controls cell behaviors such as cell proliferation, migration, apoptosis, and ECM assembly in glomerular diseases. We previously demonstrated that TGF-β1 increases not only ECM including fibronectin (FN), but also α1β1 and α5β1 integrins in rat and human GN [7,8]. In addition, we showed that both PDGF-BB and TGF-β1 are involved in ECM remodeling via β1 and α1β1 integrins in cultured MCs [9–11]. These findings suggest that cell adhesion to ECM might play an important role in the development and progression of glomerular injury. Integrin is connected with focal adhesion proteins, which include focal adhesion kinase, integrin-linked kinase, paxillin, and adaptor molecules such as tensin and talin. Focal adhesions serve as the mechanical linkages to the ECM, and as biochemical signaling hubs to concentrate and direct numerous signaling proteins at sites of integrin binding and clustering [12].

Recently, we demonstrated that hydrogen peroxide-inducible clone (Hic)-5 was localized in MCs and its expression was associated with glomerular cell proliferation and matrix expansion in human and rat GN [13]. Hic-5, a similar homologue of paxillin, is a TGF-β1- and hydrogen peroxide-inducible focal adhesion protein [14]. Hic-5 is considered to act as an adaptor molecule, and links signals from the ECM to cytoskeletal regulation and intracellular signaling which might regulate cell behavior including cell proliferation, migration, and apoptosis. It has been proposed that Hic-5 might be involved in apoptosis of MC in the development of glomerulosclerosis [15]. However, it is unclear whether Hic-5 is involved in MC proliferation as the key feature in GN. In the present study, we investigated the contribution of Hic-5 to MC proliferation in Habu venom-induced mesangioproliferative GN using Hic-5-deficient (Hic-5-/-) mice and cultured MCs isolated from Hic-5-/- mice.

## Materials and Methods

### Reagents and antibodies (Abs)

Mouse monoclonal Abs against Hic-5 (clone 34) and α-smooth muscle actin (SMA) were obtained from BD Transduction Laboratories (Franklin Lakes, NJ) and Sigma Inc. (St. Louis, MO), respectively. Rabbit monoclonal anti–Ki-67 Abs and rabbit polyclonal anti-FN, anti-cleaved caspase-3, and anti-PDGF receptor β subunit (PDGF-R-β) Abs were obtained from Millipore (Billerica, MA). Rabbit polyclonal Abs against PDGF-B chain, TGF-β1, TGF-β1 receptor type II (TGF-β-R), and cyclin A were purchased from Santa Cruz Biotechnology, Inc. (Dallas, TX). Rabbit monoclonal anti-cyclin D1 (clone EP12) Abs and mouse monoclonal anti-p21 (clone SX118) Abs were purchased from DakoCytomation (Glostrup, Denmark). Fluorescein isothiocyanate-labeled donkey anti-rabbit and anti-mouse IgGs, and rhodamine-labeled donkey anti-mouse IgG were purchased from Jackson Immuno-Research Laboratories (West Grove, PA).

### Experimental design

Hic-5-/- mice were previously generated by Dr. Kaneyama [16]. Hic-5+/+ mice (C57BL/ 6) were purchased from CLEA (Tokyo, Japan). Littermates of Hic-5-/- and Hic-5+/+ mice, which were developed following more than 12 generations of backcrosses, were used. All experimental procedures were performed according to the guidelines of the Animal Research Committee at the University of Tokushima Graduate School of Health Biosciences, and the protocol was approved by the Tokushima University Institutional Review Board for animal protection (Permit Number: 14046). All procedures were performed under enough ether anesthesia to ameliorate mice suffering. A model of mesangioproliferative GN was induced as described previously, with some modifications [6,17]. Briefly, left kidneys of 8 week-old male Hic-5+/+ and Hic-5-/- mice were removed. One week later, venom from Habu-snake (Wako, Osaka, Japan) dissolved in saline (4 mg/kg body weight) was intravenously injected through a tail vein. Heminephrectomized control mice were produced by replacing Habu venom with phosphate buffered saline. Kidney samples were harvested 7 days after injection (n = 8).

### Measurement of urinary protein excretion and serum creatinine

Urine was collected from the mice in individual metabolic cages (CL-0355; CLEA). The amount of urinary protein excretion was measured by the Bradford method (Bio-Rad, Oakland, CA). Serum creatinine was measured using Creatinine Assay Kit manufactured by BioVision (Milpitas, CA).

### Histology and immunohistochemistry

The left kidney of each mouse was removed immediately, fixed and embedded in paraffin, and 4 μm sections were stained with periodic acid-Schiff reagent. The number of glomerular cells was assessed on the basis of the total glomerular cell count per glomerular cross-section. The glomerular cell number was determined in 30 glomeruli per kidney in a periodic acid-Schiff-stained section, and the mean number of cells per glomerulus was calculated. A pathologist who was blinded to other findings semiquantitatively analyzed the matrix score. The percentage of each glomerulus that was occupied by mesangial matrix was estimated and assigned a code, as follows: 0 = absent, 0.5 = 1 to 5%; 1 = 5 to 25%; 2 = 25 to 50%; 3 = 50 to 75%; or 4 = >75%.

For immunohistochemistry, 4 μm sections were deparaffinized and rehydrated. Endogenous peroxidase was blocked before antigen retrieval by heating in 0.01 mol/l citrate buffer (pH 6.0). The sections were incubated with mouse monoclonal anti-Ki-67 Abs (1:100) and rabbit monoclonal anti-cleaved caspase-3 Abs (1:100) at 4°C for 24 h and followed by biotinylated secondary antibody and avidin-biotin-peroxidase complex (ABC Elite; Vector Laboratories, Burlingame, CA). Each section was reacted with 3,3′-diaminobenzidine (Dojindo, Kumamoto, Japan) and counterstained with Mayer’s hematoxylin (Wako), dehydrated, and coverslipped. Ki-67-positive cells were counted in 30 full-size glomeruli [18]. Negative control staining was performed by omitting primary Abs.

Immunofluorescence staining was performed using Abs against Hic-5 (1:30), α-SMA (1:50), FN (1:50), PDGF-B chain (1:30), PDGF-R-β (1:30), TGF-β1 (1:30), and TGF-β-R (1:30). The sections were incubated with an appropriate fluorescein isothiocyanate-labeled secondary antibody (1:30). The fraction of immunoreactive area (green) was measured using EIS-Elements software (Nikon Corporation, Tokyo, Japan). 30 equatorially sectioned glomeruli were examined for each section, and the positively immunoreactive glomerular area was statistically analyzed.

### Detection of apoptotic glomerular cells in GN

Apoptotic glomerular cells in GN were detected by a DeadEnd fluorometric TUNEL (terminal deoxynucleotidyl transferase dUTP nick end labeling) system following the manufacturer’s instructions (Promega, Madison, WI). Fragmented DNA was identified by incorporating fluorescein-12-dUTP at the 3′-OH DNA ends with terminal deoxynucleotidyl transferase in terminal deoxynucleotidyl transferase incubation buffer. The nuclei of the cells were counterstained with propidium iodide. Apoptotic glomerular cells were counted in 30 full-size glomeruli.

### Cultured mesangial cells isolated from Hic-5+/+ and Hic-5-/- mice

Mouse glomeruli were isolated by the perfusion of magnetic beads (Dynal Biotech ASA, Oslo, Norway) and passing through a cell strainer [19]. After the isolation of glomeruli from three mice, cultured MCs were developed and characterized according to published methods [9]. Cell morphology was observed by inverted phase-contrast microscopy CKX41 (Olympus, Tokyo, Japan). For staining MCs, cells were plated on Lab-Tek chamber slides (Thermo Fisher Scientific Inc., Waltham, MA). Cells were rinsed with phosphate buffered saline, fixed with 3% paraformaldehyde, and incubated with anti-α-SMA Abs (1:200) for 30 min at 37°C. After being washed, cells were incubated with appropriate rhodamine-conjugated secondary IgG (1:200). Cells were washed, mounted, and photographed with a Nikon fluorescence microscope (Eclipse E600).

### Cell adhesion assays

Cell adhesion assays were performed as described previously [20]. Briefly, collagen type I and FN (10 ng/mL, BD BioSciences, San Jose, CA) prepared in PBS containing 1 mM CaCl_2_ and 1 mM MgCl_2_ were coated on 96-well plates. As a control, wells were coated with 1% BSA overnight at 4°C. After being blocked with 1% BSA, 5 x 10^4^ cells were removed by dissociation solution (Sigma), washed in serum-free medium, added to each well, and the cells were allowed to adhere to the plate for 45 min at 37°C. The plate was washed with PBS containing 1 mM CaCl_2_ and 1 mM MgCl_2_ until no cells remained in the wells coated with BSA. Cell adhesion was evaluated by staining adherent cells with 0.1% crystal violet, solubilizing cells with 0.2% Triton X-100 in PBS, and reading the absorbance at 630 nm with a Microplate Reader (Bio-Rad). All samples were done in triplicate and at least three different isolations of cells were used.

### Proliferation assay

To assess cell proliferation on a 60mm culture dish, quiescent Hic-5+/+ and Hic-5-/- MCs (5x10^4^/plate) were stimulated with appropriate concentrations of PDGF-BB (50 ng/ml) and TGF-β1 (10 ng/ml) for 48 h. MCs were counted after harvesting and assessed as the mean cell number. A WST-8 assay (Dojindo) was performed to determine the number of viable cells in cell proliferation. Serum-starved MCs were incubated at 37°C at concentrations of 6x10^3^/100 μl media in triplicate in 96 well-culture plates. MCs were stimulated with PDGF-BB (50 ng/ml) and TGF-β1 (10 ng/ml) for 24 hours [21]. The stimulated MCs were pulsed with 10 μl CCK-8 solution for 3 hours, and absorbance was measured at 450 nm.

### Western blotting

MCs and kidney tissues were treated with cell lysis buffer (Cell Signaling), and protein concentrations were determined using the BCA protein assay kit (Pierce Biotechnology, Rockford, IL). Protein samples (10 μg for MCs and 30μg for kidney tissues) were separated by SDS-PAGE using a 12.5% gel and transferred to nitrocellulose membranes. The membranes were probed with either primary antibody (1:1000), followed by an appropriate HRP-conjugated secondary antibody (1:5000). Immunoreactive proteins were visualized using an enhanced chemiluminescence detection system (Amersham, Arlington Heights, IL). Blots were appropriately reproved with anti-β-actin Abs (1:10000) as a loading control. Bands were quantified by ImageJ 1.47v (National Institutes of Health; Bethesda, MD) [22].

### Statistical analysis

Values are expressed as the means ± standard deviation. Differences were evaluated with StatMate software ver 5.01 (ATMS Co., Ltd., Japan). Variables were compared between groups by one-way ANOVA and Dunnett’s test. All experiments were repeated at least three times. Values of P<0.05 were considered statistically significant [23].

## Results

### Effect of Hic-5 on MC proliferation in Habu-venom induced GN

To assess how Hic-5 affects MC proliferation in mesangioproliferative GN, we produced GN in Hic-5+/+ and Hic-5-/- mice by injecting Habu venom. As previously described in other papers, the pathological lesion of this GN was generally characterized by an initial capillary ballooning due to mesangiolysis, leading to compensatory MC proliferation and matrix expansion. This glomerular cell proliferation gradually decreased and improved to normal architecture around 4 weeks. [24,25]. Thus, we focused on the investigation of the role of Hic-5 on MC proliferation, because the expression of Hic-5 was mainly localized in MCs and enhanced during glomerular injury. Since the high dose of Habu venom (6 mg/kg body weight) was very toxic, we used the low dose of Habu venom (4 mg/kg body weight) to establish our experimental model of mice GN, which showed similar MC proliferation to rat Thy-1 GN. In this situation, both groups did not significantly show higher levels of proteinuria (1.83 ± 0.49 mg/day and 1.85 ± 0.55 mg/day in Hic-5+/+ mice vs. 1.78 ± 0.49 mg/day and 1.68 ± 0.11 mg/day in Hic-5-/- mice on day 0 and day 7, respectively) and serum creatinine (0.24 ± 0.032 mg/dl and 0.25 ± 0.036 mg/dl in Hic-5+/+ mice vs. 0.25 ± 0.017 and 0.26 ± 0.041 in Hic-5-/- mice on day 0 and day 7, respectively).

**Fig 1:**
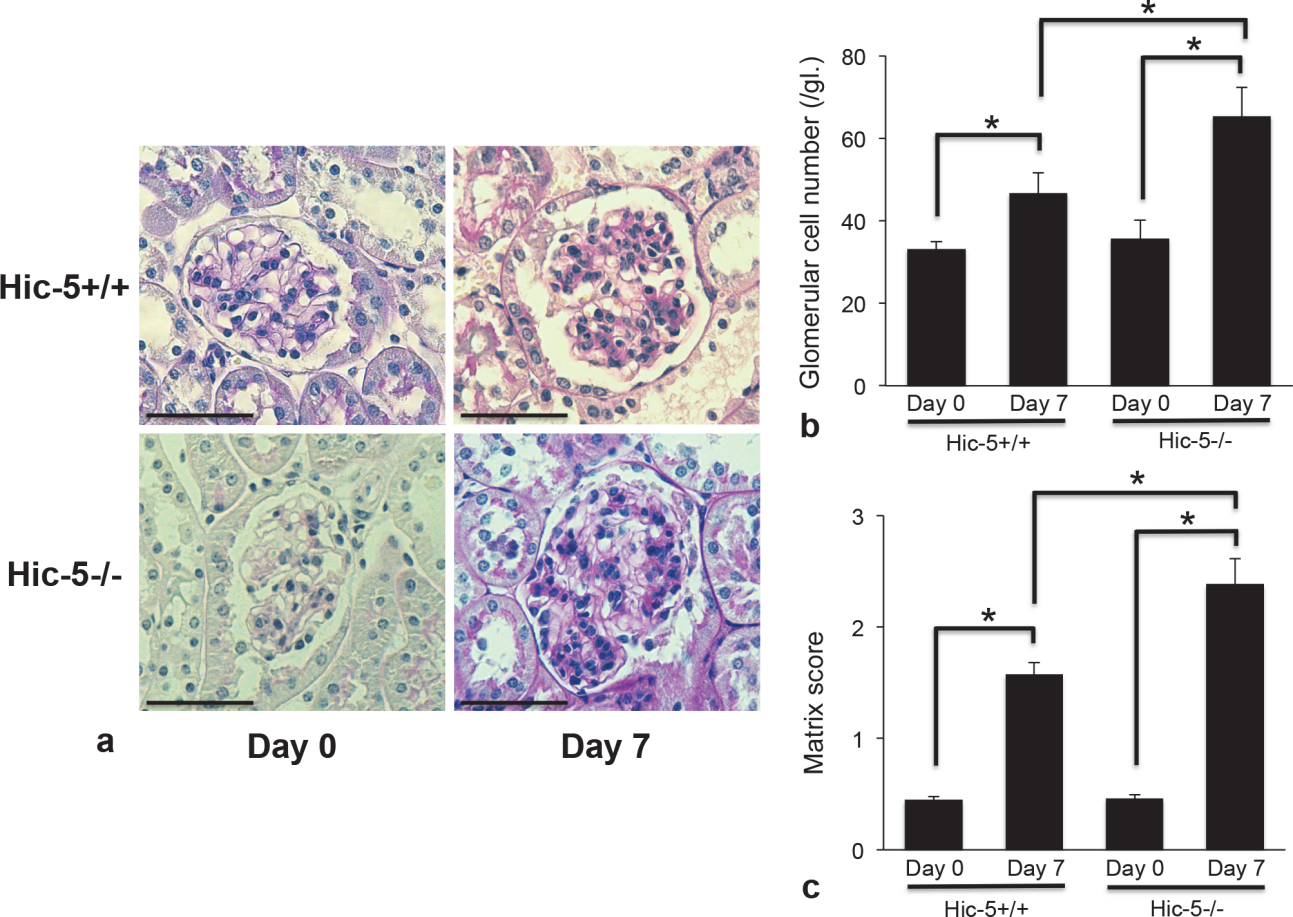
Histological assessment of glomerular lesions in experimental glomerulonephritis (GN). (a) Periodic acid-Schiff staining in Habu venom-induced Hic-5+/+ and Hic-5-/- GN mice. On day 7, Hic-5+/+ GN Habu mice exhibited glomerular cell proliferation. Hic-5-/- Habu GN mice showed severe mesangial cell proliferation and intensive glomerular matrix expansion compared to Hic-5+/+ Habu GN mice. Original magnification x200, scale bar = 50 μm. (b) The number of glomerular cells was counted in 30 glomeruli per section and calculated. The data are shown as the means ± SD. *, P<0.01. There was no significant difference between Hic-5+/+ day 0 and Hic-5-/- day 0. (c) A matrix score was assessed in 30 glomeruli per section and calculated. The data are shown as the means ± SD. *, P<0.01. There was no significant difference between Hic-5+/+ day 0 and Hic-5-/- day 0.

On day 7, Hic-5+/+ GN mice exhibited glomerular cell proliferation, and Hic-5-/- GN mice showed more severe glomerular cell proliferation and matrix expansion (Fig 1A). The glomerular cell number and matrix score in Hic-5-/- GN mice were significantly greater than those in Hic-5+/+ GN mice on day 7 (Fig 1 B and 1 C). Thereafter, glomerular cell proliferation similarly recovered around 4 weeks in both Hic-5+/+ and Hic-5-/- mice.

**Fig 2:**
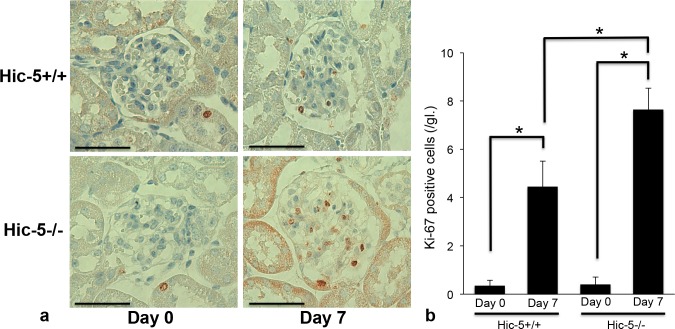
Immunohistochemistry for Ki-67 in Hic-5+/+ and Hic-5-/- glomerulonephritis (GN) mice. (a) On day 7, Hic-5+/+ Habu GN mice showed a greater number of Ki-67-positive glomerular cells. In addition, more Ki-67-positive cells were observed in the glomeruli of Hic-5-/- Habu GN mice. There were few Ki-67-positive cells in the glomeruli of Hic-5+/+ and Hic-5-/- mice on day 0. Original magnification x200, scale bar = 50 μm. (b) The number of Ki-67-positive cells was counted in 30 glomeruli per section and calculated. The data are shown as the means ± SD. *, P<0.01. There was no significant difference between Hic-5+/+ day 0 and Hic-5-/- day 0.

Glomerular Ki-67 (a marker of proliferating cells)-positive cells were significantly increased in Hic-5+/+ GN mice on day 7. The glomeruli of Hic-5-/- GN mice contained more Ki-67-positive cells than those of Hic-5+/+ GN mice on day 7 (Fig 2). On day 0, the glomeruli of Hic-5+/+ and Hic-5-/- mice contained few Ki-67-positive cells.

### Effects of Hic-5 on the expression of α-SMA and FN in Habu venom-induced GN

**Fig 3:**
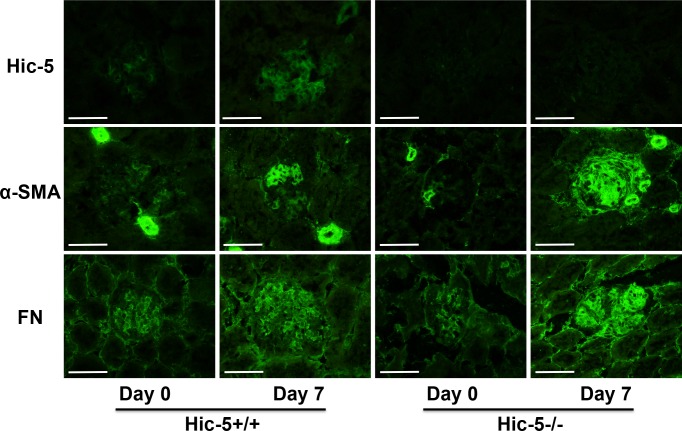
Glomerular expression of Hic-5, α-smooth muscle actin (SMA), and fibronectin (FN) in glomerulonephritis (GN) mice. Representative immunofluorescence micrographs show the glomerular expression of Hic-5, α-SMA, and FN in Hic-5+/+ and Hic-5-/- GN mice on day 0 and day 7. Original magnification x200, scale bar = 50 μm.

**Fig 4:**
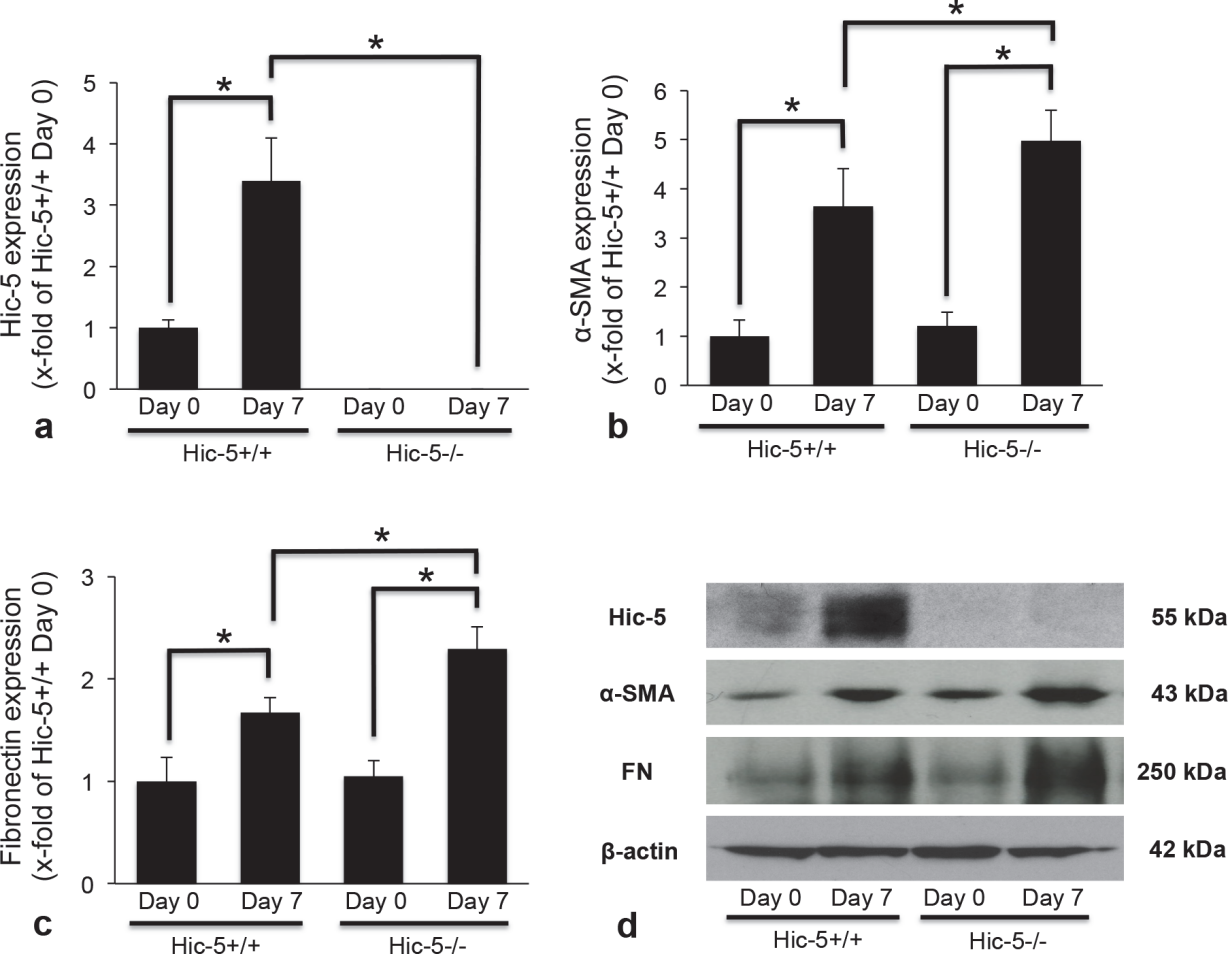
Semi-quantitative assessment of the glomerular expression of Hic-5, α-smooth muscle actin (SMA), and fibronectin (FN). The glomerular expression of Hic-5 (a), α-SMA (b), and FN (c) in Hic-5+/+ and Hic-5-/- glomerulonephritis mice was determined as the positively immunoreactive fraction in the glomerular area based on the examination of 30 equatorially sectioned glomeruli for each section, and statistically analyzed. The data are shown as the means ± SD. *, P<0.01. There was no significant difference in the glomerular expression of α-SMA and FN between Hic-5+/+ day 0 and Hic-5-/- day 0, respectively. (d) The expression of Hic-5, α-SMA, and FN in kidney cortical tissues in GN was also investigated by western blotting. Expression of β-actin is shown as a loading control. Each experiment was performed at least three times.

We previously demonstrated that the expression of α-SMA, a marker of activated MC, was increased and associated with Hic-5 expression in diseased glomeruli in human and rat mesangioproliferative GN [13]. Thus, we investigated the possibility that α-SMA expression could be affected by the absence of Hic-5 expression in Hic-5-/- GN mice, since MCs are activated and show α-SMA-positive myofibroblastic characteristics in GN. Hic-5 expression was increased in glomeruli of Hic-5+/+ GN mice on day 7. Negative Hic-5 staining was confirmed in Hic-5-/- GN mice on days 0 and 7 (Fig 3 and 4A). α-SMA expression was very weak in glomeruli of Hic-5+/+ and Hic-5-/- mice on day 0. On day 7, α-SMA expression in glomeruli of Hic-5+/+ GN mice was increased. α-SMA expression in glomeruli of Hic-5-/- GN mice was greater than that in glomeruli of Hic-5+/+ GN mice (Fig 3 and 4B). These results suggest that MCs might be highly activated after glomerular injury in Hic-5-/- GN mice.

To examine the effect of Hic-5 on ECM accumulation, immunofluorescence staining was performed with anti-FN Abs. FN expression was weak in glomeruli of Hic-5+/+ and Hic-5-/- mice on day 0. On day 7, this expression was highly increased and accompanied by matrix expansion in glomeruli of Hic-5+/+ GN mice. Glomerular FN expression in glomeruli of Hic-5-/- GN mice was greater than that in glomeruli of Hic-5+/+ GN mice on day 7 (Fig 3 and 4C). These results suggest that FN expression might be associated with MC proliferation and ECM accumulation in GN. These immunofluorescence data were furthermore confirmed by the western blotting using kidney cortical tissues in GN (Fig 4D).

### Effects of Hic-5 on the expression of PDGF-B chain, PDGF-B receptor, TGF-β1, and TGF-β receptor in Habu venom-induced GN

**Fig 5:**
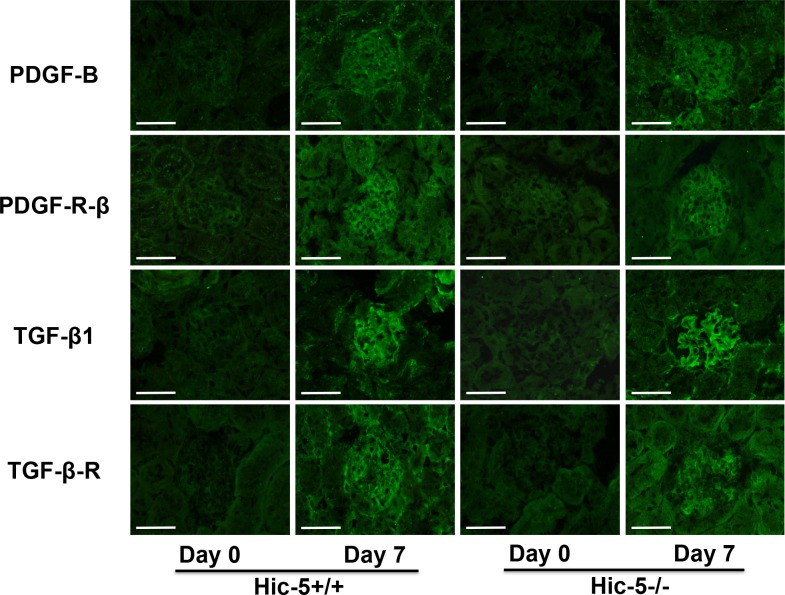
Glomerular expression of PDGF-B chain, PDGF receptor, TGF-β1, and TGF-β receptor in glomerulonephritis (GN) mice. Representative immunofluorescence micrographs show the glomerular expression of PDGF-B chain, PDGF-receptor β subunit (PDGF-R-β), TGF-β1, and TGF-β receptor type II (TGF-β-R) in Hic-5+/+ and Hic-5-/- GN mice on day 0 or day 7. Original magnification x200, scale bar = 50 μm.

**Fig 6:**
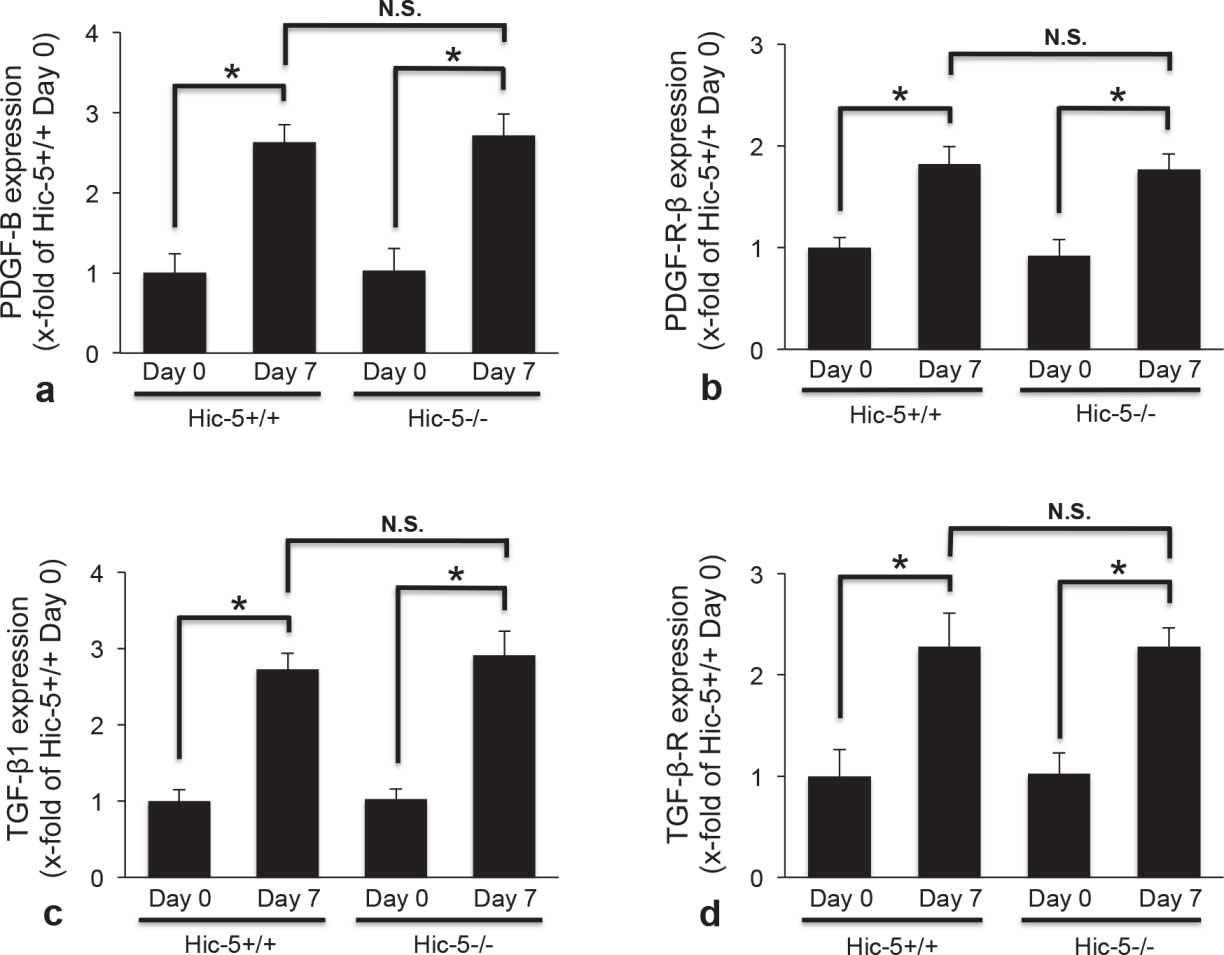
Semi-quantitative assessment of the glomerular expression of PDGF-B chain, PDGF receptor, TGF-β1, and TGF-β receptor. The glomerular expression of PDGF-B chain (a), PDGF receptor β subunit (PDGF-R-β) (b), TGF-β1 (c), and TGF-β receptor type II (TGF-β-R) (d) in Hic-5+/+ and Hic-5-/- glomerulonephritis mice was determined as the positively immunoreactive fraction in the glomerular area based on the examination of 30 equatorially sectioned glomeruli for each section, and statistically analyzed. The data are shown as the means ± SD. *, P<0.01. N.S., not significant. There was no significant difference between Hic-5+/+ day 0 and Hic-5-/- day 0.

Since the increased MC proliferation and ECM accumulation in diseased glomeruli are affected by growth factors, we investigated the glomerular expression of PDGF-B chain and TGF-β1, and their receptors, against PDGF and TGF-β. The expression of both PDGF-B chain and TGF-β1 was weak in glomeruli of Hic-5+/+ and Hic-5-/- mice on day 0. On day 7, the expression was enhanced to a similar degree in glomeruli of Hic-5+/+ and Hic-5-/- GN mice. In addition, the expression of receptors against PDGF and TGF-β was similar to the expression of PDGF-B chain and TGF-β1 (Fig 5). Assessment by analyzing positively immunoreactive glomerular area showed that there were no significant differences between Hic-5+/+ and Hic-5-/- GN mice on days 0 and 7, respectively. These results suggested that Hic-5 did not affect the glomerular expression of PDGF-B chain, TGF-β1, or their receptors before or after glomerular injury (Fig 6).

### Effect of Hic-5 on glomerular cell apoptosis in Habu venom-induced GN

**Fig 7:**
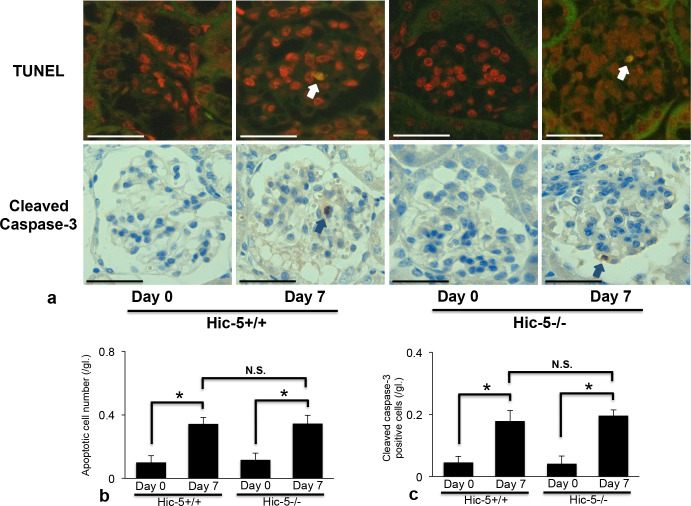
Detection of apoptotic glomerular cells in Hic-5+/+ and Hic-5-/- glomerulonephritis (GN) mice. (a) Representative TUNEL (terminal deoxynucleotidyl transferase dUTP nick end labeling) staining (upper) and cleaved caspase-3 positive cells (lower). Apoptotic glomerular cells in GN were identified by fluorescence-labeled nuclei (yellow), indicated by arrows. The nuclei of glomerular cells were counterstained with propidium iodide (red). In addition, cleaved caspase-3 positive cells were detected by immunohistochemistry and indicated by arrows. Original magnification x200, scale bar = 50 μm. (b) The number of apoptotic glomerular cells was counted and calculated in 30 full-size glomeruli. (c) The number of cleaved caspase-3 positive cells was counted and calculated in 30 full-size glomeruli. The data are shown as the means ± SD. *, P<0.01. N.S., not significant. There was no significant difference between Hic-5+/+ day 0 and Hic-5-/- day 0 on both apoptotic glomerular cells and cleaved caspase-3 positive cells.

Since Hic-5 might be involved in the apoptosis of MC in the development of glomerulosclerosis [15], a TUNEL assay was performed to examine apoptotic cells in glomeruli of Hic-5+/+ and Hic-5-/- mice. There are few apoptotic cells in glomeruli of Hic-5+/+ and Hic-5-/- mice on day 0. On day 7, apoptotic cells were slightly but significantly increased in glomeruli of Hic-5+/+ and Hic-5-/- GN mice compared to glomeruli of control mice. In addition, there were no significant differences between Hic-5+/+ and Hic-5-/- GN mice on day 7 (Fig 7 A and 7 B).

To assess whether caspase-3 is activated for glomerular cell apoptosis in GN, cleaved caspase-3 positive cells were detected in GN glomeruli by immunohistochemistry. There are few cleaved caspase-3 positive cells in glomeruli of Hic-5+/+ and Hic-5-/- mice on day 0. On day 7, cleaved caspase-3 positive cells were slightly but significantly increased in glomeruli of Hic-5+/+ and Hic-5-/- GN mice compared to glomeruli of control mice. In addition, there were no significant differences between Hic-5+/+ and Hic-5-/- GN mice on day 7 (Fig 7 A and 7 C).

### Effect of Hic-5 on the proliferation of cultured MCs isolated from Hic-5+/+ and Hic-5-/- mice

**Fig 8:**
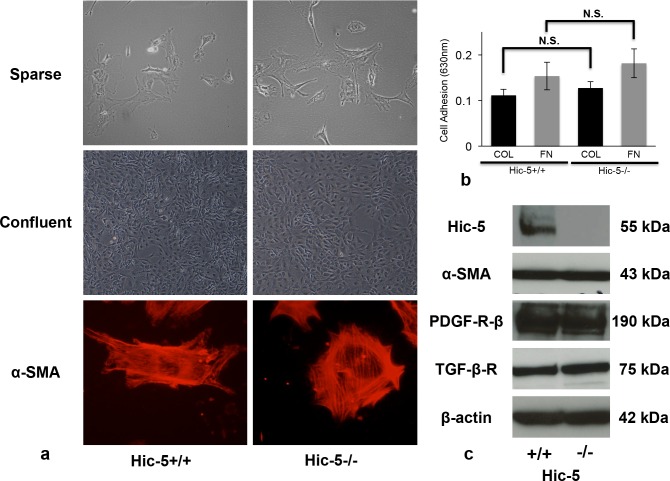
Morphology and characterization of cultured mesangial cells (MCs) isolated from Hic-5+/+ and Hic-5-/- mice. (a) MCs isolated from both Hic-5+/+ and Hic-5-/- mice were cultured after the isolation of glomeruli using magnetic beads. Cell morphology is shown in sparse (upper panel) and confluent (middle panel) conditions. Representative images were captured by an inverted microscope (Olympus CKX41). Original magnification x100 (upper) and x40 (middle), respectively. Lower panels show rhodamine α-smooth muscle actin (SMA) staining by fluorescence microscopy (Nikon Eclipse E600). Original magnification x400. (b) MC adhesion to collagen type I (COL:10 ng/mL) and fibronectin (FN:10 ng/mL) were shown as absorbance (630 nm). The data are shown as the means ± SD. N.S., not significant.

To examine whether Hic-5 controls MC proliferation in vitro, we developed cultured MCs after the isolation of mice glomeruli [19]. The cell phenotype was morphologically similar between Hic-5+/+ and Hic-5-/- MCs under regular conditions (Fig 8A). However, microscopic observation of cells stained with anti-α-SMA Abs showed that α-SMA formed a longitudinal pattern in approximately 87.3% of Hic-5+/+ MCs, and a peripheral pattern in approximately 80.3% of Hic-5-/- MCs (Fig 8A). This phenotypic difference was supported by a previous report that Hic-5 knockdown induced an amoeboid phenotype that exhibited reduced plasticity in breast cancer cell lines [26]. To examine whether Hic-5 affects adhesive potential to ECM, cell adhesion assays to collagen type I and FN were performed. Adhesion to these ECMs showed no significant difference, suggesting that cell adhesive property would not affect MC proliferation (Fig 8B).

To investigate the effects of the representative growth factors PDGF-BB and TGF-β1, we first confirmed that both Hic-5+/+ and Hic-5-/- MCs expressed similar levels of PDGF-R-β and TGF-β-R (Fig 8C). These data suggested that stimulation with PDGF-BB and TGF-β1 should affect both cells to a similar degree. Thus, we assessed MC proliferation under the addition of PDGF-BB and TGF-β1.

**Fig 9:**
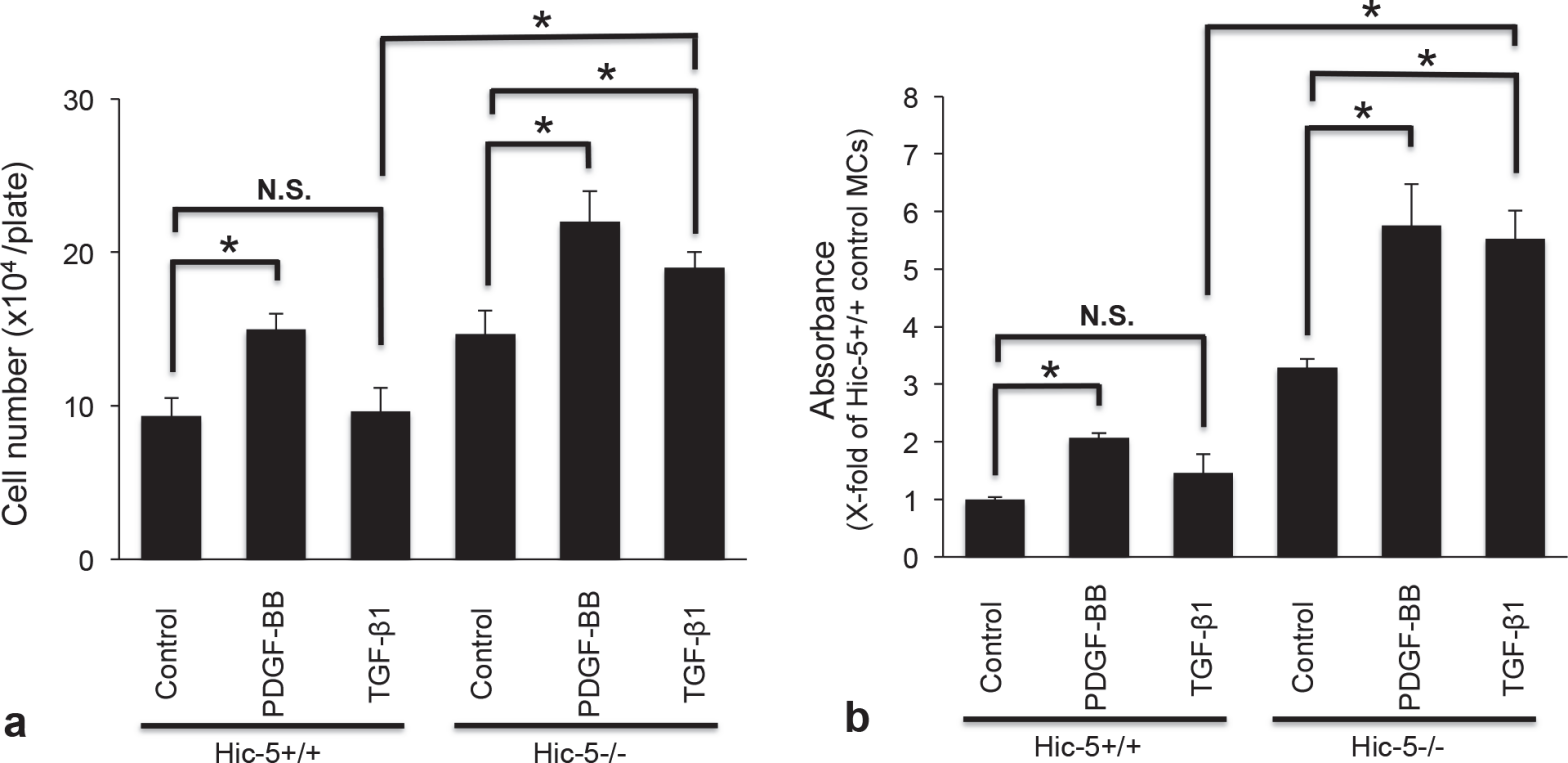
Assessment of cell proliferation in cultured mesangial cells (MCs) isolated from Hic-5+/+ and Hic-5-/- mice. (a) Quiescent Hic-5+/+ and Hic-5-/- MCs (5x10^4^/plate) were stimulated with appropriate concentrations of PDGF-BB (50 ng/ml) and TGF-β1 (10 ng/ml) for 48 h. MCs were counted after harvesting and assessed as the mean cell number. The data are shown as the means ± SD. *, P<0.01. N.S., not significant. Control Hic-5-/- MCs showed a significantly increased cell number compared to control Hic-5+/+ MCs (P<0.01). (b) Quiescent MCs (6x10^3^/100 μl) in 96-well culture plates were stimulated with PDGF-BB (50 ng/mL) or TGF-β1 (10 ng/mL) for 24 hours. The stimulated MCs were pulsed with 10 μl CCK-8 solution for 3 hours, and absorbance was measured at 450 nm. The data are shown as the means ± SD. *, P<0.01. Control Hic-5-/- MCs showed significantly increased absorbance compared to control Hic-5+/+ MCs (P<0.01). Each experiment was performed independently a minimum of three times.

A cell-counting assay showed that there were slightly more Hic-5-/- MCs than Hic-5+/+ MCs. PDGF-BB (50 ng/ml) increased Hic-5-/- MCs more than Hic-5+/+ MCs for 48 hours. TGF-β1 (10 ng/ml) significantly increased the number of Hic-5-/- MCs, but not Hic-5+/+ MCs (Fig 9A). In addition, we performed a WST-8 assay, which determines cell viability in cell proliferation. This assay demonstrated that Hic-5-/- MCs showed greater absorbance than Hic-5+/+ MCs in the absence of growth factors. While PDGF-BB (50 ng/ml) increased absorbance in both Hic-5+/+ and Hic-5-/- MCs, absorbance in the latter was greater than that in the former. TGF-β1 (10 ng/ml) produced a significant increase in the absorbance of Hic-5-/- MCs, but not Hic-5+/+ MCs (Fig 9B). These results suggested that Hic-5-/- MCs have mitogenic potential compared to Hic-5+/+ MCs in the presence and absence of growth factors.

### Effect of Hic-5 on the expression of cyclins A and D1, and the cyclin-dependent kinase inhibitor protein p21 by cultured MCs

**Fig 10:**
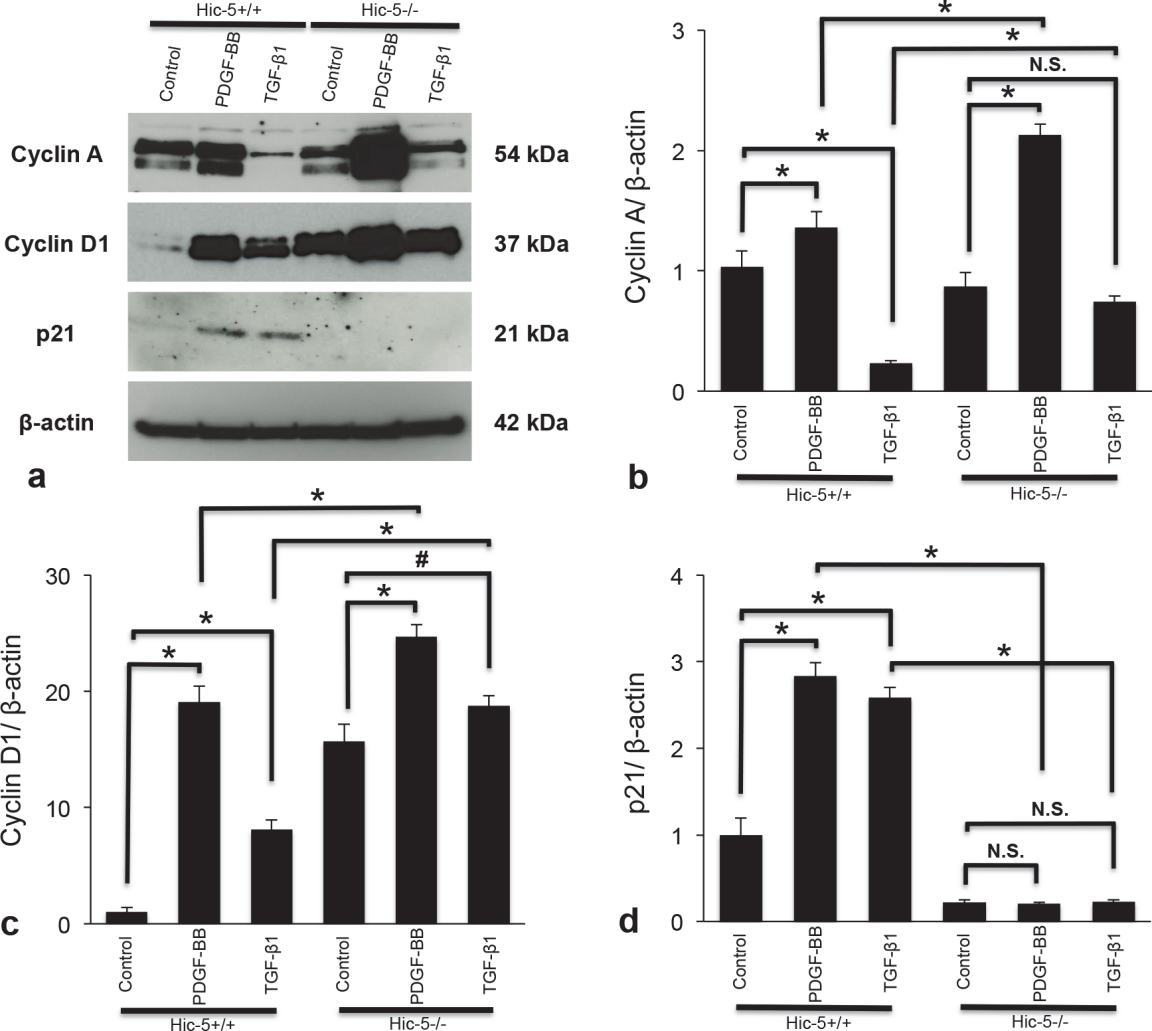
Expression of cyclins A and D1, and p21 in cultured mesangial cells (MCs). (a) Representative western blotting images show levels of cyclins A and D1, and p21 in Hic-5+/+ and Hic-5-/- MCs with or without stimulation by PDGF-BB (50 ng/ml) or TGF-β1 (10 ng/ml). Expression of β-actin is shown as a loading control. Quantitative data obtained by ImageJ 1.47v (National Institutes of Health) are shown as the means ± SD. The bar graphs b, c, and d indicate the expression of cyclin A, cyclin D1, and p21, respectively. *, P<0.01. #, P<0.05. N.S., not significant. The results shown are representative of three independent experiments. There were significant differences in the expression of cyclin D1 and p21 between control Hic-5+/+ and Hic-5-/- MCs without stimulation (P<0.01). There was no significant difference in the expression of cyclin A between control Hic-5+/+ and Hic-5-/- MCs without stimulation.

To examine the effects of Hic-5 and growth factors on the cell cycle, the expression of cyclins A and D1, and p21 was detected by western blot analysis (Fig 10). Incubation of Hic-5+/+ MCs with PDGF-BB (50 ng/ml) markedly increased the protein levels of cyclins A and D1 in the cells. In contrast, TGF-β1 (10 ng/ml) decreased cyclin A expression, and upregulated cyclin D1 in Hic-5+/+ MCs. Quiescent Hic-5-/- MCs showed a greater expression of cyclin D1 than Hic-5+/+ MCs without stimulation. PDGF-BB strongly enhanced the expression of cyclins A and D1 in Hic-5-/- MCs compared to Hic-5+/+ MCs. In addition, TGF-β1 enhanced the expression of cyclins A and D1 in Hic-5-/- MCs compared to Hic-5+/+ MCs. p21 expression was increased in Hic-5+/+ MCs stimulated with PDGF-BB or TGF-β1. In contrast, p21 expression by PDGF-BB or TGF-β1 was not detected in Hic-5-/- MCs (Fig 10). These results suggested that Hic-5 might be involved in the regulation of MC proliferation through the altered and coordinated expression of cyclins A and D1, and p21.

## Discussion

MC proliferation and ECM accumulation, representative pathological features, are observed during the progression of GN. In general, MC proliferation precedes and is tightly linked to ECM accumulation and the development of glomerulosclerosis. Since a therapeutic strategy to reduce MC proliferation also decreases ECM accumulation in GN, many researchers have focused on MC proliferation and its critical mechanisms [27–29].

We previously demonstrated that Hic-5 expression was enhanced in diseased glomeruli of human and rat mesangioproliferative GN and its expression was associated with MC proliferation and ECM accumulation [13]. However, the role that Hic-5 plays in MC proliferation and ECM accumulation in GN is still unclear. To assess the role of Hic-5 in MC proliferation, we used mice with deletion of the Hic-5 gene and developed an animal model of mesangioproliferative GN by the injection of Habu venom after heminephrectomy. Hic-5-/- mice showed no obvious phenotypic change or abnormal developmental events in any organs including the kidneys, as previously described under normal conditions [16]. Once glomerular injury was induced, the numbers of glomerular cells and Ki-67-positive proliferating cells in Hic-5-/- GN mice were significantly greater than those in Hic-5+/+ GN mice. These results suggested that Hic-5 might prevent the acceleration of MC proliferation in Habu venom-induced glomerular injury.

Based on previous studies on representative growth factors, PDGF-BB is considered to be a potent stimulator of MC proliferation, whereas TGF-β1 controls the growth factor-induced mitogenic MC response [6]. The expression of both PDGF-BB and TGF-β1 was increased in human GN as well as in the acute phase of mesangioproliferative GN including anti-Thy1 GN [8,30–33]. Habu venom-induced GN after the removal of one kidney also showed reproducible glomerular cell proliferation similar to that observed in the acute phase of GN as in anti-Thy1 GN. On day 7, the expression of PDGF-B chain and TGF-β1 was increased in diseased glomeruli of Hic-5+/+ and Hic-5-/- GN mice. The glomerular expression of receptors for PDGF-B chain and TGF-β was also increased in Hic-5+/+ and Hic-5-/- GN mice on day 7, which was supported by previous research [30–32,34,35]. Notably, there were no significant differences in the expression of PDGF-B chain, TGF-β1, or their receptors between Hic-5+/+ and Hic-5-/- GN mice on day 7. These results suggest that these representative growth factors affect glomerular cells after glomerular injury to a similar degree. Thus, in vivo experiments indicate that Hic-5 itself has the potential to regulate MC proliferation, since Hic-5 deletion appeared to directly affect the increase in the number of glomerular cells in Hic-5-/- GN mice. Interestingly, Hic-5 overexpressing osteoblast clones showed a decrease in proliferation, that supports our present study [36]. In addition, our culture experiment showed that PDGF-BB induced a greater proliferation of Hic-5-/- MCs than Hic-5+/+ MCs, suggesting that the mitogenic potential of PDGF-BB is partially regulated by Hic-5 expression. In contrast, TGF-β1 significantly stimulated Hic-5-/- MC proliferation, but not Hic-5+/+ MC proliferation. With regard to the inhibition of MC proliferation by TGF-β1, Schöcklmann *et al*. reported that TGF-β1 induced cell cycle arrest in MCs by inhibiting cyclin E-cdk 2 activation and retinoblastoma protein phosphorylation [37]. Kitamura and Suto also stated that TGF-β1 might inhibit the mitogenic responses of MCs by suppressing inflammatory cytokines from macrophages and modulating the cell-cycle machinery of glomerular cells [38]. Interestingly, Dabiri *et al*. reported that Hic-5 slows the growth of myofibroblasts from a hypertrophic skin scar where its expression is upregulated by the autocrine induction of TGF-β1 [39]. Although the key molecules by which TGF-β1 controls MC growth have not yet been identified, Hic-5 might be responsible for the TGF-β1-inducible regulation of MC growth.

An increase in the number of glomerular cells might be due to decreased apoptosis in diseased glomeruli of Hic-5-/- GN mice, since a change in the glomerular cell number is based on the balance between proliferation and apoptosis [1]. Hornigold *et al*. reported that Hic-5 mediates the susceptibility of MC to apoptosis in a rat remnant kidney model and therefore is involved in the development of glomerulosclerosis [15]. MC apoptosis is important for the progression and resolution of mesangioproliferative GN, however, we could not detect a significant difference in apoptosis between Hic-5+/+ and Hic-5-/- GN mice on day 7 in the present study. Thus, Hic-5 might preferentially affect MC proliferation rather than MC apoptosis in the acute mitogenic phase in these GN model mice.

Several studies have indicated that the cell cycle plays a role in cell behavior including proliferation and apoptosis through cell attachment. Schöcklmann previously demonstrated that the protein expression of cyclins D1 and E was markedly downregulated in MCs plated on polymerized collagen type I, suggesting that ECM might regulate MC proliferation via integrins [40]. In addition, our previous work demonstrated that overexpression of α1β1 integrin, one of the major collagen receptors on MC, was associated with the increased expression of p27 and regulation of MC proliferation [10]. In the present study, western blotting data supported the notion that Hic-5 expression also regulates MC proliferation via the modulation of cyclins and a cyclin-dependent kinase inhibitor. PDGF-BB or TGF-β1 might enhance MC proliferation in Hic-5-/- MCs through the altered and coordinated expression of cyclins A and D1, and p21. Thus, Hic-5 expression might interfere with MC proliferation through regulation of the cell cycle under not only TGF-β1 stimulation but also MC adhesion to ECM protein via integrins and focal adhesion.

In conclusion, the present study suggested that physiological or controlled expression of Hic-5 plays a role in managing the number of glomerular cells after glomerular injury. Hic-5 suppresses MC proliferation in the acute mitogenic phase of GN. However, there is limitation to define whether Hic-5 affects the outcome of chronic glomerular diseases including IgA nephropathy and diabetic nephropathy in the present study. To clarify whether modulation of Hic-5 expression become a novel therapeutic maneuver to prevent the progression of chronic GN, further additional researches are required.
